# RNAMethyPro: a biologically conserved signature of N6-methyladenosine regulators for predicting survival at pan-cancer level

**DOI:** 10.1038/s41698-019-0085-2

**Published:** 2019-05-01

**Authors:** Raju Kandimalla, Feng Gao, Ying Li, Hao Huang, Jia Ke, Xin Deng, Linjie Zhao, Shengtao Zhou, Ajay Goel, Xin Wang

**Affiliations:** 1 Center for Gastrointestinal Research; Center for Translational Genomics and Oncology, Baylor Scott & White Research Institute and Charles A Sammons Cancer Center, Baylor University Medical Center, Dallas, TX USA; 2 Department of Biomedical Sciences, City University of Hong Kong, Hong Kong, China; 3 The Sixth Affiliated Hospital of Sun Yat-Sen University, Guangzhou, China; 4 Department of Obstetrics and Gynecology, Key Laboratory of Birth Defects and Related Diseases of Women and Children of MOE and State Key Laboratory of Biotherapy, West China Second University Hospital, Sichuan University and Collaborative Innovation Center, Chengdu, China; 5 Shenzhen Research Institute, City University of Hong Kong, Hong Kong, China

**Keywords:** Prognostic markers, Tumour biomarkers

## Abstract

Accumulating evidence indicates the role of *N*^6^-methyladenosine (m^6^A) regulator-mediated RNA methylation in cancer progression and metastasis; yet its potential clinical significance, if any, remains unclear. In this first-of-its-kind study, we systematically evaluated the role of m^6^A regulators as potential disease biomarkers based on comprehensive analysis of gene expression profiles of 9770 cancer cell lines and clinical specimens from 25 publicly available datasets, encompassing 13 human cancers. We developed and established RNAMethyPro—a gene expression signature of seven m^6^A regulators, which robustly predicted patient survival in multiple human cancers. Pan-cancer analysis identified activated epithelial–mesenchymal transition (EMT), as a highly conserved pathway in high-risk patients predicted by RNAMethyPro in 10 of the 13 cancer types. A network-based analysis revealed an intimate functional interplay between m^6^A regulators and EMT-associated factors via druggable targets such as XPO1 and NTRK1. Finally, the clinical significance of RNAMethyPro was further exemplified in colorectal cancer, where high-risk patients demonstrated strong associations with a mesenchymal subtype, activated stromal infiltration, and poor therapeutic response to targeted anti-EGFR therapy. In summary, RNAMethyPro is a novel, EMT-associated prognostic gene-expression signature in multiple human cancers and may offer an important clinical decision-making tool in the future.

## Introduction

Among >100 types of known posttranscriptional modifications, *N*^6^-methyladenosine (m^6^A) represents the most prevalent internal modification in mammalian mRNAs,^1^ which is primarily predominant in the vicinity of stop codons, 3′-untranslated regions (UTRs), within long internal exons, and at 5′-UTRs.^2–4^ These m^6^A modifications are posttranscriptionally installed, erased, and recognized by m^6^A *writers* [METTL3, METTL14 (methyltransferase-like 3, 14) and WTAP1 (Wilms’ tumor 1-associating protein)],^5–7^ *erasers* [FTO (fat mass and obesity-associated protein), ALKBH5 (alkylated DNA repair protein AlkB homolog 5)]^1,8,9^ and *readers* [YTHDF1, YTHDF2, and YTHDF3 (YTH *N*^6^-Methyladenosine RNA Binding Protein)],^10–12^ respectively. The functional consequence of such m^6^A modifications includes reduced RNA stability, translational inefficiency, altered subcellular localization, and imperfect alternate splicing.^10,13,14^ While low m^6^A levels maintain the cells in a state of pluripotency, their overexpression results in cellular differentiation, suggesting their potential role in the establishment of a “stem cell phenotype” in human cancer.^15^

Recent functional studies in glioblastoma (GBM), breast cancer, hepatocellular carcinoma (HCC), lung cancer, and acute myeloid leukemia (AML), involving either the knockdown or overexpression of m^6^A methyl transferases (METTL3, METTL14) or demethylases (FTO, ALKBH5), have revealed their critical biological role in driving cellular proliferation, migration, invasion, apoptosis, and metastasis.^16–19^ In addition, low expression of METTL14 in HCC^20^ and overexpression of FTO in breast and gastric cancer has been shown to associate with poor prognosis.^21,22^ Interestingly, MLL-rearranged leukemic subtype and HER2-overexpressing breast cancer subtypes associated with upregulation of FTO,^23^ indicating the role of these genes in driving poor prognosis-related molecular subtypes in these malignancies.

Although studies to date have provided important insights into the role of m^6^A regulators in cancer pathogenesis, these efforts have heavily relied on the use of cancer cell lines and/or small cohorts of patient specimens, making them unreliable for fully appreciating their clinical significance. For instance, *METTL3* and *METTL14* were shown to be oncogenic in AML^24–26^ but tumor suppressive in GBM.^16^ Curiously, even for the same cancer type (e.g., GBM), the role of the same gene (e.g., *METTL3*) was reported to be discordant in independent studies.^16,27^ These studies highlight the imperative need for undertaking systematic, large-scale studies in independent patient cohorts to unravel the true clinical potential of m^6^A regulators in human cancers.

Herein, using a systematic, pan-cancer approach, we developed RNAMethyPro, a novel and robust gene expression signature based upon m^6^A regulators, for predicting the prognosis of patients in 13 different human cancer types. Interestingly, RNAMethyPro not only allowed identification of high-risk cancer patients with poor prognosis but also led to the recognition that de-regulated expression of m^6^A-regulators was intimately associated with an epithelial–mesenchymal transition (EMT) phenotype, which was highly conserved across ten cancer types. More specifically, in colorectal cancer (CRC) patients, RNAMethyPro-led identification of the high-risk group significantly associated with the mesenchymal subtype, demonstrated activation of EMT and transforming growth factor beta (TGFβ) pathway, increased cancer stemness and higher overall stromal and immune content. Further a network-based analysis suggested strong physical and functional crosstalk between m^6^A machinery and key EMT-associated proteins such as XPO1 and NTRK1—for which therapeutic interventions have already been approved by the Food and Drug Administration (FDA) or are currently being explored in various clinical trials. In addition to its prognostic utility, RNAMethyPro also emerged as a robust predictor of response to anti-epidermal growth factor receptor (anti-EGFR) therapy in colorectal patients with metastatic disease. Taken together, our findings provide compelling data for the clinical significance of m^6^A regulators and set the stage for future validation and further in-depth mechanistic studies in future.

## Results

### A panel of seven m^6^A regulator genes predicts patient survival in various cancers

**Fig. 1:**
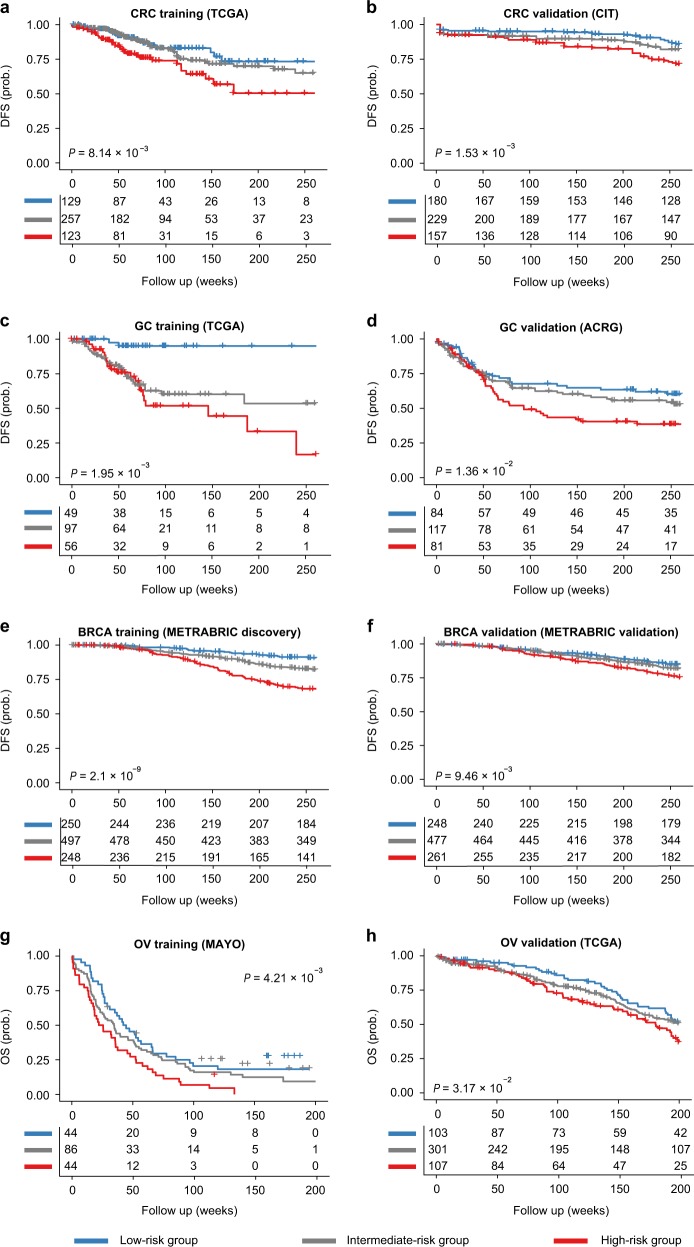
Internal and external validations in colorectal cancer (CRC), gastric cancer (GC), breast cancer (BRCA) and ovarian cancer (OV) demonstrated the robust prognostic value of RNAMethyPro. Kaplan–Meier curves show significant associations between RNAMethyPro stratified risk groups and disease-free survival or overall survival for **a** CRC training cohort, TCGA-COADREAD (*n* = 509); **b** CRC validation cohort, CIT (*n* = 566); **c** GC training cohort, TCGA-STAD (*n* = 202); **d** GC validation cohort, ACRG-GC (*n* = 282); **e** BRCA training cohort, METABRIC discovery (*n* = 995); **f** BRCA validation cohort, METABRIC validation (*n* = 986); **g** OV training cohort, MAYO-OV (*n* = 174); **h** OV validation cohort, TCGA-OV (*n* = 511). Only patients with available survival information were included in the analyses. Patients classified to RNAMethyPro high-, intermediate- and low-risk groups are colored in red, gray, and blue, respectively

We systematically evaluated the prognostic significance of m^6^A regulatory machinery, focusing on a panel of 3 m^6^A “writers” (METTL3, METTL14 and WTAP), 2 “erasers” (FTO and ALKBH5), and 2 “readers” (YTHDF1 and YTHDF2). We performed comprehensive bioinformatics analysis of 25 public gene expression datasets comprising a total of >9000 patients across 13 cancer types (Table 1, Supplementary Material and Methods). For each type of cancer, a multivariate Cox regression model was first trained using the corresponding training dataset, and the derived formula (hereafter referred to as “RNAMethyPro”) was subsequently used to calculate risk scores predictive of overall survival (OS; for ovarian and pancreatic cancer) or relapse-free survival (for the other 11 cancer types). Using cutoff thresholds on the 25th and 75th percentiles of the risk scores, patients in each cohort were stratified into low-, intermediate-, and high-risk groups. We observed that the high-risk patients had a significantly shorter survival compared to low-risk patients (Fig. 1a, c, e, g, Supplementary Fig. S1, Table 2), indicating that the prognostic power of RNAMethyPro was successfully validated in all the 13 cancer types.

**Table 1:** A summary of public datasets analyzed in this study

Cancer type	Internal/external validation	Cohort name	Data source	Platform	Sample type	# of samples	PubMed ID	Used for GSEA
Colorectal cancer	Internal	TCGA-COADREAD	TCGA	Illumina GA/HiSeq RNA-Seq	Fresh frozen	626	22810696	Y
	External	CIT	GSE39582	Affymetrix Human Genome U133 Plus 2.0 Array	Fresh frozen	566	23700391	
	CRC Meta-validation cohort	Jorissen	GSE14333	Affymetrix Human Genome U133 Plus 2.0 Array	Fresh frozen	290	19996206	
		Smith	GSE17536	Affymetrix Human Genome U133 Plus 2.0 Array	Fresh frozen	177	19914252	
		Birnbaum	GSE26906	Affymetrix Human Genome U133 Plus 2.0 Array	Fresh frozen	86	22496922	
		AMC-AJCCII-90	GSE33113	Affymetrix Human Genome U133 Plus 2.0 Array	Fresh frozen	90	22056143	
		Laibe	GSE37892	Affymetrix Human Genome U133 Plus 2.0 Array	Fresh frozen	130	22917480	
		Kirzin	GSE39084	Affymetrix Human Genome U133 Plus 2.0 Array	Fresh frozen	68	25083765	
		Khambata-Ford	GSE5851	Affymetrix Human Genome U133 Plus 2.0 Array	Biopsies	80	17664471	
		Medico	GSE59857	Illumina HumanHT-12 V4.0 expression beadchip	Cell line	155	25926053	
		Barretina	CCLE	Illumina HiSeq RNA-Seq	Cell line	58	22460905	
Gastric cancer	Internal	TCGA-STAD	TCGA	Illumina HiSeq RNA-Seq	Fresh frozen	415	25079317	Y
	External	ACRG-GC	GSE62254	Affymetrix Human Genome U133 Plus 2.0 Array	FFPE	300	25894828	
Breast cancer	Internal	METABRIC Discovery	METABRIC	Illumina HT-12 v3	Fresh frozen	997	22522925	
	External	METABRIC Validation	METABRIC	Illumina HT-12 v3	Fresh frozen	995	22522925	
		TCGA-BRCA	TCGA	Illumina HiSeq RNA-Seq	Fresh frozen	1100	26451490	Y
Ovarian cancer	Internal	MAYO-OV	GSE53963	Affymetrix HG133A microarray	Fresh frozen	174	25269487	Y
	External	TCGA-OV	TCGA	Illumina HiSeq RNA-Seq	Fresh frozen	514	21720365	
Lung squamous cell carcinoma	Internal	TCGA-LUSC	TCGA	Illumina HiSeq RNA-Seq	Fresh frozen	501	22960745	Y
Hepatocellular carcinoma	Internal	TCGA-LIHC	TCGA	Illumina HiSeq RNA-Seq	Fresh frozen	373	28622513	Y
Head and neck squamous cell carcinoma	Internal	TCGA-HNSC	TCGA	Illumina HiSeq RNA-Seq	Fresh frozen	522	25631445	Y
Esophageal squamous cell carcinoma	Internal	TCGA-ESCA	TCGA	Illumina HiSeq RNA-Seq	Fresh frozen	92	28052061	Y
Esophageal adenocarcinoma	Internal				Fresh frozen	73		Y
Lung adenocarcinoma	Internal	TCGA-LUAD	TCGA	Illumina HiSeq RNA-Seq	Fresh frozen	517	25079552	Y
Bladder urothelial carcinoma	Internal	TCGA-BLCA	TCGA	Illumina HiSeq RNA-Seq	Fresh frozen	408	24476821	Y
Pancreatic adenocarcinoma	Internal	TCGA-PAAD	TCGA	Illumina HiSeq RNA-Seq	Fresh frozen	179	28810144	Y
Acute myeloid leukemia	Internal	TARGET-AML	TARGET	Illumina HiSeq RNA-Seq	Blood	284	26941285	Y
Total						9770

**Table 2:** Log-rank test and univariate analysis of RNAMethyPro risk score in each cohort analyzed for internal or external validation

Cancer type	Cohort	*P* value^a^	HR^a^ (95% CI)	*P* value^b^	*P* value of risk score^c^
Colorectal cancer	TCGA-COADREAD	8.14E−03	2.15 (1.20–33.83)	9.25E−03	3.06E−03
Colorectal cancer	CIT	1.53E−03	2.24 (1.34–3.74)	4.68E−03	4.17E−03
Gastric cancer	TCGA-STAD	1.95E−05	11.98 (2.81–51.07)	2.41E−04	3.22E−05
Gastric cancer	ACRG-GC	1.36E−02	1.78 (1.12–2.83)	3.83E−02	3.23E−03
Breast cancer	METABRIC Discovery	2.10E−09	3.96 (2.43–6.44)	2.04E−09	1.09E−09
Breast cancer	METABRIC Validation	9.46E−03	1.73 (1.14–2.63)	2.17E−02	3.95E−03
Ovarian cancer	MAYO-OV	4.21E−03	1.91 (1.22–2.99)	1.24E−02	1.81E−03
Ovarian cancer	TCGA-OV	3.17E−02	1.56 (1.04–2.35)	8.22E−02	5.37E−01
Pancreatic adenocarcinoma	TCGA-PAAD	2.19E−03	4.48 (1.59–12.65)	6.62E−03	2.80E−03
Hepatocellular carcinoma	TCGA-LIHC	4.36E−04	2.25 (1.42–3.57)	1.19E−04	4.27E−06
Lung adenocarcinoma	TCGA-LUAD	4.97E−04	2.51 (1.47–4.28)	3.97E−04	4.25E−05
Bladder urothelial carcinoma	TCGA-BLCA	2.83E−04	3.08 (1.63–5.84)	1.32E−03	4.73E−03
Head and neck squamous cell carcinoma	TCGA-HNSC	2.18E−07	3.27 (2.04–5.24)	1.42E−07	2.35E−05
Acute myeloid leukemia	TARGET-AML	1.32E−04	2.2 (1.45–3.32)	1.29E−04	1.99E−06
Lung squamous cell carcinoma	TCGA-LUSC	2.35E−02	4.79 (1.07–21.42)	5.00E−02	2.26E−02
Esophageal adenocarcinoma	TCGA-ESCA(EAC)	1.78E−02	NA^d^	5.51E−02	5.31E−03
Esophageal squamous cell carcinoma	TCGA-ESCA(ESCC)	1.25E−02	6 (1.23–29.31)	5.48E−02	1.39E−02

*CI* confidence interval, *HR* hazard ratio, *NA* not applicable

^a^Log-rank test (high-risk vs low-risk groups)

^b^Log-rank test (three groups)

^c^Univariate Cox regression

^d^HR cannot be accurately estimated owing to insufficient sample size

For four cancer types (colorectal, gastric, breast, and ovarian) where additional independent patient cohorts were available, we next sought to externally validate the prognostic potential of RNAMethyPro. For CRC, the risk scoring formula trained using the TCGA-COADREAD cohort was subsequently applied to the CIT cohort (*n* = 566), followed by stratification of the patients based by applying the same cutoff thresholds determined in the training cohort. Consistent with the TCGA-COADREAD cohort, in the CIT cohort, we also observed that the high-risk patients had a significantly shorter disease-free survival (DFS) vs low-risk patients (*P* = 0.00153, log-rank test) with a corresponding hazard ratio (HR) of 2.24 (1.34–3.74; Fig. 1b, Table 2). Similarly, the m^6^A signature showed robust potential for predicting survival in validation cohorts in gastric (Fig. 1d, ACRG-GC cohort: HR, 1.78 [1.12–2.83], *P* = 0.0136), breast (Fig. 1f, METABRIC validation cohort: HR, 1.73 [1.14–2.63], *P* = 0.00946), and ovarian cancer (Fig. 1h, TCGA-OV cohort: HR, 1.56 [1.04–2.35], *P* = 0.0317). Taken together, by using systematic statistical approaches on both the internal and external validation cohorts, we were able to demonstrate the robust prognostic significance of RNAMethyPro in various cancers.

### Identification of highly conserved biological processes associated with cancer metastasis in high-risk patients identified by RNAMethyPro

**Fig. 2:**
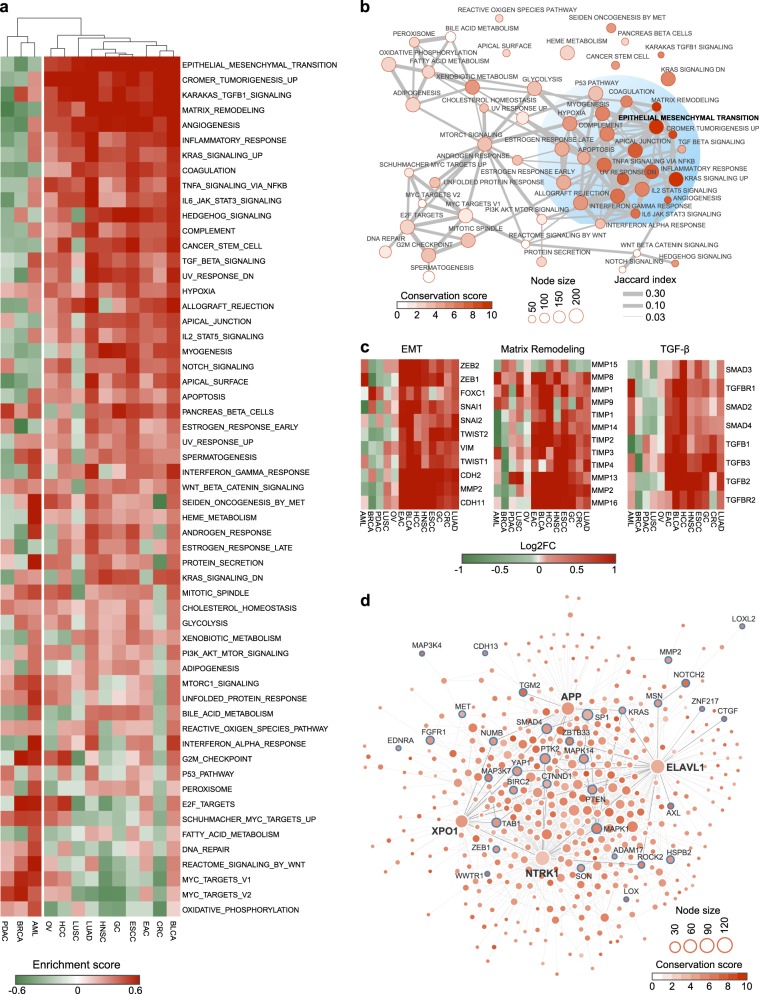
Pan-cancer functional analyses identified conserved biological processes and protein–protein interaction (PPI) subnetwork dysregulated in RNAMethyPro high-risk patients. **a** Heatmap of enrichment scores of hallmark gene sets across 13 cancer types. Hierarchical clustering on the enrichment score matrix identified a small cluster consisting of BRCA, AML, and PDAC and a major cluster of the other ten cancer types. **b** An enrichment map illustrating associations between hallmark gene sets with different degrees of conservation across various cancer types. Node size represents the number of genes in a gene set. Nodes are colored in proportion to the conservation scores of gene sets across ten cancer types of the major cluster. Edges between gene sets showed their association quantified by Jaccard index. To make the network relatively sparse, edges with extremely low Jaccard indices (<0.03) were removed. **c** Heatmaps showing the average log2 fold difference of the indicated genes (rows) in core gene sets for epithelial–mesenchymal transition (EMT), matrix remodeling, and transforming growth factor-β pathway between RNAMethyPro high- and low-risk groups across the 13 different cancer types. **d** Conserved PPI subnetwork underlying the RNAMethyPro high-risk patients across the 10 cancer types identified using BioNet (false discovery rate <1e−4). Node size is proportionate to the degree of each node in the network. Node color represents the conservation score calculated by the number of times (out of the total 10 cancer types) that the corresponding gene is differentially expressed in the high-risk group compared to the low-risk group (Benjamini–Hochberg adjusted *P* < 0.05). Nodes with labels represent EMT signature genes. Hub proteins (NTRK1, XPO1, ELAVL1, and APP) in the network are highlighted with bold labels. Edges represent physical PPIs between genes obtained from BioGRID database (version 3.4.134). Edges colored in dark black represent interactions between the four hub proteins and EMT signature gene products

To gain insight into the mechanistic underpinnings of high-risk patients identified by RNAMethyPro, we systematically interrogated various key biological processes dysregulated across the 13 cancer types. More specifically, for each cancer type, we analyzed the corresponding gene expression datasets (Table 1) for gene set enrichment analysis (GSEA) on 50 hallmark gene sets obtained from MSigDB using HTSanalyzeR.^28^ Unsupervised hierarchical clustering on the obtained matrix of gene set enrichment scores identified two distinct clusters of cancers—a small cluster comprising of breast (BRCA), pancreatic (PDAC), and acute myeloid leukemia (AML) and a major cluster of ten other cancer types. Interestingly, the major cluster was primarily enriched for gastrointestinal (GI) cancers typified by specific biological processes related to EMT, angiogenesis, and cancer stemness (Fig. 2a). Interestingly, different from other GI cancers, activation of MYC and pancreatic beta cells emerged as major drivers of disease pathogenesis in PDAC.^29–31^ Breast cancer patients with poor prognosis were characterized by basal subtype-specific features such as MYC and E2F activation,^32^ whereas high-risk AML subgroup associated with heme metabolism and interferon-alpha response, in line with previous reports.^33,34^

To further dissect the biological properties associated with RNAMethyPro high-risk groups, we constructed a comprehensive enrichment map and identified a subnetwork of highly conserved biological processes associated with cancer progression and metastasis (Fig. 2b). Central to this functional network of pathways was EMT, which was significantly upregulated in the RNAMethyPro high-risk group in all the ten cancer types within the major cluster (Supplementary Fig. S2). Core signature genes for EMT, matrix remodeling processes, and TGF-β were mostly significantly upregulated in RNAMethyPro-identified high-risk patients in all GI cancers (except PDAC) and lung adenocarcinoma (LUAD; Fig. 2c). Interestingly, lung squamous cell carcinoma (LUSC), which is another major type of non-small-cell lung carcinoma, did not show any significant upregulation of these signature genes in the RNAMethyPro high-risk subgroup (Fig. 2c)—highlighting the specificity of our m^6^A signature for different cancer types.

To identify functionally conserved modules underlying the dysregulated biological processes associated with the RNAMethyPro high-risk groups, we employed a network-based approach by integrating human interactome and gene expression data. Interestingly, the conserved subnetwork of protein–protein interactions (PPIs) we identified were enriched for a number of EMT signature genes (Fig. 2d). Central to the network were four hub proteins including, APP,^35^ XPO1,^36^ NTRK1,^37^ and ELAVL1 (or HuR),^38^ which have been previously implicated for their regulatory roles in tumorigenesis and/or metastasis. Taken together, our findings revealed that upregulation of EMT is a key common mechanism associated with high-risk cancer patients, highlighting potential interactions between m^6^A regulatory machinery and cancer metastasis.

### The RNAMethyPro high-risk group in CRC associates with the mesenchymal subtype

**Fig. 3:**
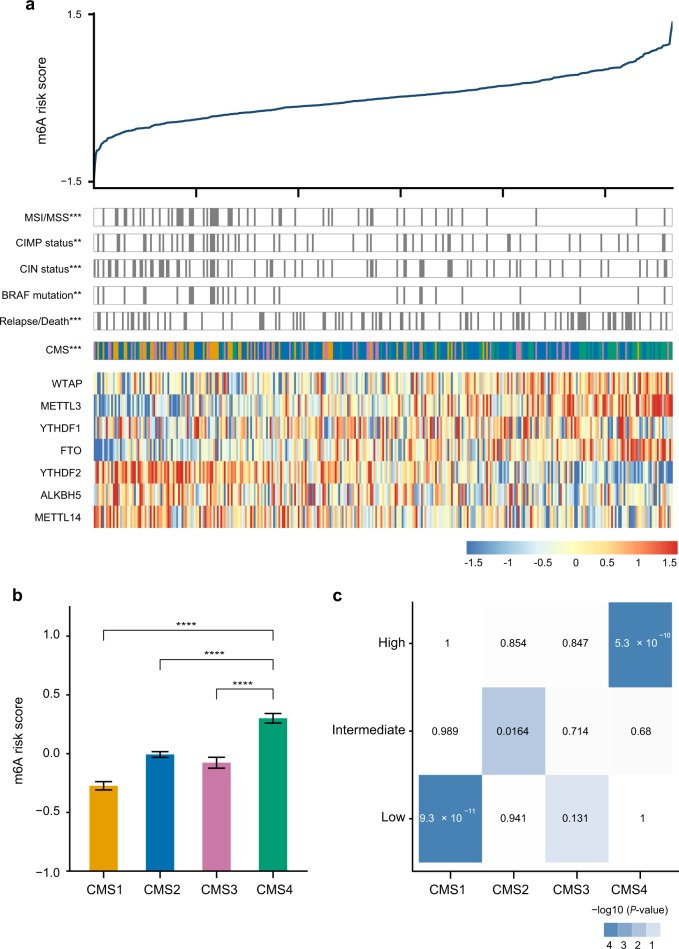
RNAMethyPro high-risk group is significantly associated with the mesenchymal subtype of colorectal cancer (CRC0. **a** Significant associations were found between the RNAMethyPro risk groups (high vs low risk) and clinical and molecular characteristics in the CIT cohort (**P < 0.01, and ***P < 0.001, Fisher’s exact tests). Heatmap shows the expression levels of m^6^A signature genes in all patient samples ordered by RNAMethyPro risk score. **b** Bar plot compares RNAMethyPro risk scores of tumors classified to different consensus molecular subtypes (CMSs) in the CIT cohort. CMS4 tumors show significantly higher risk scores than CMS1, CMS2, and CMS3 (****P < 0.0001, one-tailed Student’s *t* tests. Error bar: standard error of the mean. *n* = 83 for CMS1, *n* = 225 for CMS2, *n* = 66 for CMS3, and *n* = 92 for CMS4). **c** Heatmap showing the pair-wise associations between RNAMethyPro risk groups (rows) and CMS subtypes (columns) in the CIT cohort. Colors are proportionate to the −log10-transformed *P* values derived from hypergeometric tests

By using CRC as a case study, we next performed integrative analysis to further elucidate the biological and clinical characteristics associated with the RNAMethyPro risk groups. Using TCGA-COADREAD dataset, we first trained a multivariate Cox regression model and obtained the following risk scoring formula: 0.24 × *METTL3* − 0.14 × *METTL14* + 0.09 × *WTAP* − 0.14 × *YTHDF1* − 0.22 × *YTHDF2* + 0.22 × *FTO* + 0.03 × *ALKBH5*. Based on this formula, we calculated risk scores and stratified patients in the CIT cohort (*n* = 566) using the 25th and 75th percentiles in the training cohort patients into low-, intermediate-, and high-risk groups. Interestingly, we found that the high-risk group was significantly enriched for patients with cancer relapse or death (*P* = 0.00095, Fisher’s exact test), while the low risk group significantly comprised of patients with CIN, CIMP, MSI, and *BRAF* mutations (*P* = 0.00034, 0.0063, 8.59e−11, 0.0013, respectively, Fisher’s exact tests; Fig. 3a). Notably, we found that both the low- and high-risk groups were significantly associated with unique consensus molecular subtypes (CMSs) previously defined by the CRC subtyping consortium (CRCSC)^39^ (Fig. 3a, *P* < 1e-16, Fisher’s exact test). More specifically, CMS4 patients had the highest risk scores, while CMS1 subgroup had the lowest, and CMS2 and CMS3 patients possessed in between risk scores (Fig. 3b). Hypergeometric tests further confirmed that the RNAMethyPro high-, intermediate- and low-risk groups were significantly overrepresented for patients classified to CMS4, CMS2, and CMS1, respectively (Fig. 3c, *P* = 5.30e−10 and 9.95e−08). These results are consistent with previously reported findings that patients with CMS1 tumors had the best prognosis, while CMS4 tumors resulted in the worst DFS.^39^ Furthermore, we found that indeed the RNAMethyPro high-risk group showed significant upregulation in gene sets related to the EMT, matrix remodeling, TGFβ pathway, and cancer stem cell, with concurrent downregulation of the WNT signaling pathway, MYC targets, and mesenchymal–epithelial transition (Supplementary Fig. S3), which were described as the key molecular characteristics of CMS4 CRCs.^39^

### Integrative analysis revealed complex physical and functional crosstalk between m^6^A regulators and EMT in CRC

For a better understanding of the biological processes associated with RNAMethyPro high-risk groups specifically in CRC, we systematically analyzed gene expression data for CRC cell lines from the CCLE cohort (*n* = 58) and patients from CRC Meta-validation cohort (*n* = 841),^40^ which was generated by merging six independent public datasets (Table 1). Cell lines classified to the high-risk group showed in general higher expression levels of 11 EMT signature genes than those classified to the low-risk group (Supplementary Fig. S4a). GSEA confirmed significant enrichment of EMT hallmark genes (in total 200 genes in the EMT hallmark gene set of MSigDB database) in CRC cell lines classified to the high-risk group (Supplementary Fig. S4b, *P* < 0.001). More strikingly, in the CRC Meta-validation cohort patients classified to the high-risk group had significantly higher expression levels of all EMT signature genes (Supplementary Fig. S4c, *P* < 0.05 in all comparisons, one-tailed Student’s *t* tests). Similarly, significant enrichment of EMT hallmark genes was also observed in patients classified to the high-risk group (Supplementary Fig. S4d, *P* < 0.001).

**Fig. 4:**
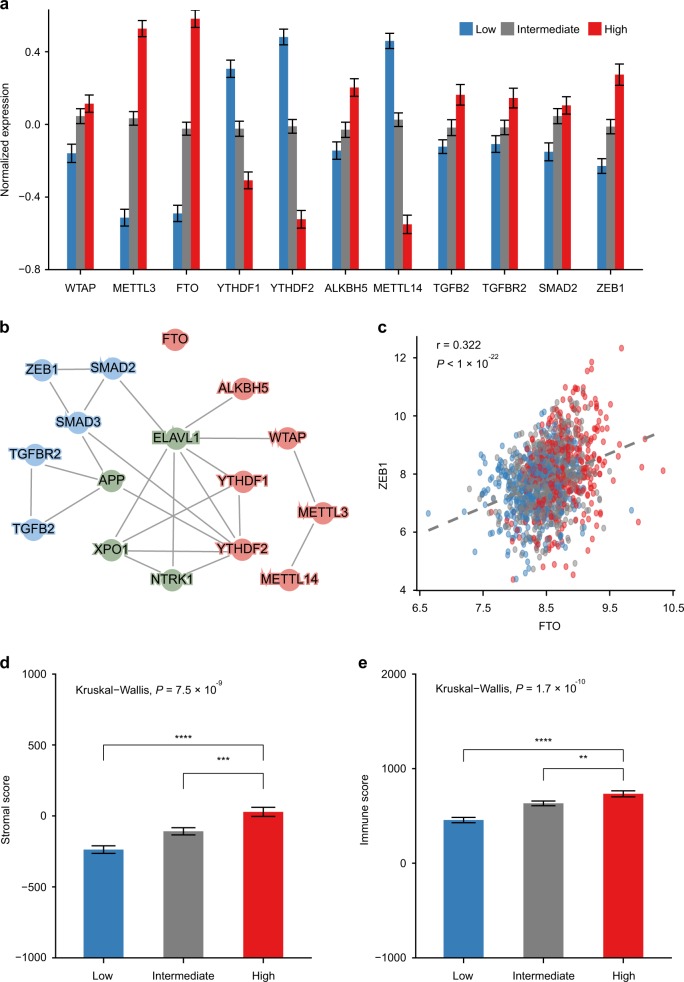
Integrative analysis revealed complex physical and functional interactions between m^6^A regulators and epithelial–mesenchymal transition (EMT). **a** Bar plot compares normalized expression levels of m^6^A regulators and EMT signature genes, showing significant differences between RNAMethyPro high- and low-risk groups (*P* < 0.01 in all comparisons, Wilcoxon rank-sum tests). **b** Protein–protein interactions between m^6^A regulators (red nodes), hub proteins in the conserved subnetwork (green nodes), and EMT key factors (blue nodes). **c** Scatter plot showing significant Pearson correlations between the *FTO* and *ZEB1* expression in CRC Meta-validation cohort (*r* = 0.322, *P* < 1e−22). Dots in the scatter plots are colored by RNAMethyPro risk groups. **d**, **e** Bar plots illustrate stromal and immune scores in CRC Meta-validation cohort calculated by ESTIMATE, indicating stronger **d** stromal and **e** immune infiltration in the RNAMethyPro high-risk group (*P* < 0.001, Kruskal–Wallis test. **P < 0.01, ***P < 0.001, ****P < 0.0001, one-tailed Student’s *t* tests). Error bar: standard error of the mean. *n* = 445 for the low-risk group, *n* = 563 for the intermediate-risk group, *n* = 398 for the high-risk group

Interestingly, among all the seven m^6^A regulators studied, *WTAP*, *METTL3*, *FTO*, and *ALKBH5* were all significantly upregulated in the high-risk group vis-à-vis low and intermediate groups (Fig. 4a, *P* < 0.001, Student’s *t* tests), while *YTHDF1*, *YTHDF2*, and *METTL14* were all significantly downregulated in the high-risk group in the CRC Meta-validation cohort (Fig. 4a, *P* < 0.001, Student’s *t* tests). Based on the observation of upregulated EMT (Supplementary Fig. S3) and associated key signature genes such as *TGFB2*, *TGFBR2*, *SMAD2*, and *ZEB1* (Fig. 4a) in the high-risk patients, we infer that m^6^A regulatory machinery must interact with EMT to regulate cancer metastasis in various human malignancies.

To systematically investigate any potential physical and functional crosstalk, we constructed a PPI network based on BioGRID database (Fig. 4b) and a coexpression network (Supplementary Fig. S5), which involved EMT signature genes, m^6^A regulators, and the four hub genes in the conserved subnetwork described earlier (Fig. 2d). Interestingly, in the PPI network, we found direct interaction between YTHDF2 and SMAD3 (Fig. 4b), in addition to the recently identified interaction between SMAD2/3 and METTL3-METTL14-WTAP complex induced by TGFβ signaling.^41^ More strikingly, most m^6^A regulators directly or indirectly interacted with the EMT gene products via hub proteins such as ELAV1 and APP (Fig. 4b). Although FTO was not found to physically interact with the EMT machinery, its gene expression was significantly correlated with *ZEB1* (Pearson correlation coefficient: 0.323, *P* = 3.55e−15), as well as *SMAD3*, *TGFB2*, and *TGFBR2* (Fig. 4c, Supplementary Fig. S5, Supplementary Table S1). Besides *FTO*, other m^6^A regulators were also intimately interconnected with hub genes in the conserved subnetwork and EMT signature genes (Supplementary Fig. S5), highlighting their intensive functional crosstalk in mediating cancer metastasis. Furthermore, compared to the RNAMethyPro intermediate- and low-risk groups, we also observed significantly higher stromal and immune infiltration (Fig. 4d, e) in the high-risk group, which is consistent with recent studies that poor prognosis CRC is a primarily a consequence of abundant stromal content with TGFβ activation.^42,43^

### RNAMethyPro is predictive of therapeutic response to anti-EGFR drugs in CRC

**Fig. 5:**
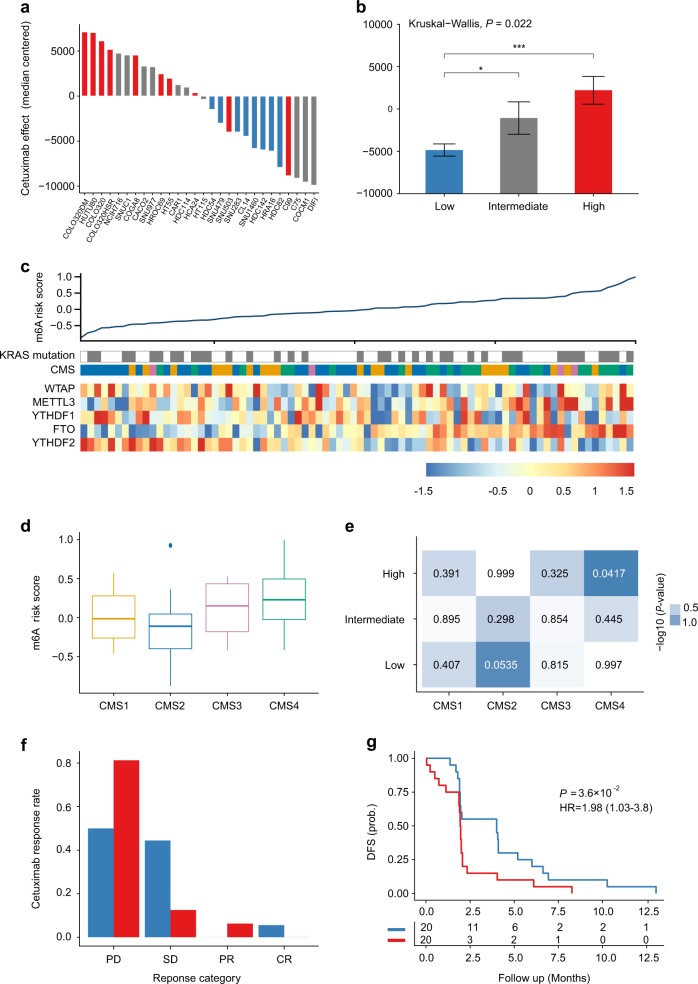
RNAMethyPro is predictive of anti-epidermal growth factor receptor therapy response in CRC cell lines and metastatic patients. **a** Waterfall plot comparing cetuximab sensitivities of 28 MSS cell lines without *KRAS*, *NRAS*, *BRAF*, and *PIK3CA* mutations.^45^ Bars represent arbitrary indices of cetuximab effects (median-centered) on cell lines as described in ref. ^45^ Cell lines sensitive to cetuximab are shown with a negative index. Cell lines classified to RNAMethyPro high-, intermediate-, and low-risk groups are colored in red, gray, and blue, respectively. **b** Barplot showing that cell lines belonging to the RNAMethyPro low-risk group are significantly more sensitive to cetuximab than those classified to the intermediate- and high-risk groups (*P < 0.05 and ***P < 0.001, one-tailed Student’s *t* tests. Error bar: standard error of the mean. *n* = 8 for the low-risk group, *n* = 10 for the intermediate-risk group, *n* = 10 for the high-risk group.). **c** Heatmap showing the expression levels of m^6^A signature genes and *KRAS* mutations in the Khambata–Ford cohort with 80 patients with metastatic cancer, ordered by RNAMethyPro risk score. **d** Boxplot showing that RNAMethyPro risk scores are significantly higher in CMS4 tumors than in non-CMS4 tumors (*P* = 0.00065, one-tailed Student’s *t* test. The median was marked as the center line in each box, the box ends indicate 25th and 75th quantiles, and the whiskers extending from the box represent 1.5 interquartile ranges of the samples. **e** Heatmap showing pairwise associations between the RNAMethyPro risk groups and consensus molecular subtype (CMS) subtypes, colored by −log10-transformed *P* values derived from hypergeometric tests. **f** Bar plot illustrating the difference in Cetuximab response between the RNAMethyPro high- and low-risk groups (*P* = 0.06, Fisher’s exact test, PD vs SD/PR/CR). CR complete response, PR partial response, SD stable disease, PD progressive disease. **g** Kaplan–Meier graph of patients stratified for RNAMethyPro risk (high-risk group vs low-risk group, *P* = 0.036, log-rank test)

Molecular subtypes of CRC are associated with response to anti-EGFR therapies independent of *KRAS* mutations.^44^ In this study, we were able to demonstrate that the RNAMethyPro risk groups were significantly associated with various CRC subtypes and accordingly hypothesized that risk scores derived from this signature may also be predictive of therapeutic response to anti-EGFR drugs. To validate our hypothesis, we first analyzed a public cohort of 151 CRC cell lines with gene expression and cetuximab sensitivity data (GSE59857).^45^ To avoid any potential confounding factors, we focused on 28 microsatellite stable cell lines without *KRAS*, *NRAS*, *HRAS*, *BRAF*, and *PIK3CA* mutations, which have been shown to be significantly associated with refractory cetuximab response.^46^ Using the established scoring formula for CRC, we calculated risk scores followed by stratification of all cell lines into low-, intermediate-, and high-risk groups. Meanwhile, based on arbitrary indices of cetuximab effect (median-centered, as described previously^45^), all cell lines could also be successfully classified into cetuximab-resistant and -sensitive groups. Indeed, we found that the predicted RNAMethyPro risk was significantly associated with cetuximab resistance (Fig. 5a, *P* = 0.00086, Fisher’s exact test). More specifically, cell lines classified into the low-risk group were significantly more resistant to cetuximab than those in the intermediate- and high-risk groups (Fig. 5b, *P* < 0.05 and *P* < 0.001, one-tailed Student’s *t* tests).

To further investigate the predictive potential of RNAMethyPro, we classified 80 metastatic CRC patients treated with cetuximab in the Khambata–Ford cohort^47^ into low-, intermediate-, and high-risk groups. Similar to the CIT cohort with mostly stage II/III patients, we observed that in the Khambata–Ford cohort CMS4 tumors also had higher risk scores compared to non-CMS4 tumors (*P* = 0.0018, one-tailed Student’s *t* test, Fig. 5d), and the high-risk group was significantly associated with CMS4 CRC subtype (*P* = 0.0417, hypergeometric test, Fig. 5e). Compared to the low-risk group, we found the high-risk group of patients may be more resistant to cetuximab treatment (progressive disease vs stable disease/partial response/complete response, *P* = 0.06, Fisher’s exact test, Fig. 5f) and were associated with significantly poorer DFS (HR 1.98, [1.03–3.80], *P* = 0.036, log-rank test, Fig. 5g). Interestingly, univariate and multivariate Cox regression analysis showed that RNAMethyPro-derived risk scores were significantly associated with poor DFS (*P* = 0.0373 and 0.0295, respectively, Supplementary Table S2), whereas *KRAS* mutation, a well-established determinant of anti-EGFR drug response, failed to show any significance (*P* = 0.213 and 0.177, respectively, Supplementary Table S2). Collectively, these results also highlight the additional potential for using RNAMethyPro as a tool for predicting therapeutic response to anti-EGFR therapy, which will refine and further optimize treatment decision-making in metastatic CRC patients.

## Discussion

Earlier studies have revealed the critical role of m^6^A regulators, particularly METTL3, METTL14, FTO and ALKBH5 in driving cancer progression and metastasis. In many cancers, m^6^A modifications can also be disrupted by genetic variants, and bioinformatic tools, represented by m6ASNP,^48^ have been developed for identification of genetic variants that target m^6^A modification sites. However, to the best of our knowledge, to date there are no systematic studies that have comprehensively analyzed the true clinical potential of the expression levels of m^6^A regulator genes in clinical decision-making. Here we have performed the most comprehensive pan-cancer analysis on the role of m^6^A regulators in multiple cancer types. The overall strengths of our study include: (1) analysis of data from >9700 cell lines and clinical specimens encompassing 13 cancer types, which represents thus far the most comprehensive analysis in the field to date; (2) the use of a network-based pan-cancer analysis to identify key pathways and protein subnetworks associated with m^6^A deregulation; (3) integrative analysis of gene expression, molecular, and clinicopathological characteristics, as well as drug response data, demonstrating the very first associations between m^6^A modifications and clinical outcomes in proof-of-principle analysis in CRC.

Our identification for the promising clinical significance of m^6^A regulators motivated us to dissect the underlying functional determinants that are potentially shared across multiple cancer types. Based on the GSEA and conservation enrichment map, we identified that biological processes such as EMT, angiogenesis, and cancer stemness were commonly upregulated in RNAMethyPro-identified high-risk patients across ten different cancers. Although the association between m^6^A regulators and EMT was proposed previously, our findings for the first time highlight this to be a key shared pathway that is highly conserved in multiple major malignancies. Interestingly, our network analysis identified a conserved functional module of protein-protein interactions enriched for EMT signature gene products, which further led us to identify four hub proteins, APP, ELAVL1 (HuR), XPO1, and NTRK1, whose roles in predicting adjuvant therapy benefit, cancer progression and metastasis have been suggested previously.^35–38^ More importantly, our discovery for the strong functional and physical interactions between these four hub proteins, m^6^A regulators and EMT signature genes suggests that the m^6^A machinery facilitates the EMT process directly or indirectly via these hub proteins in various human cancers. Furthermore, the identification of the hub proteins is clinically relevant, since they are druggable and several inhibitors are already approved by the US FDA (e.g., Entrectinib targeting NTRK1) or are currently being evaluated in clinical trials (e.g., KPT-330 targeting XPO1). Based on the observation that key EMT drivers such as *SMAD2*, *SMAD3*, *ZEB1*, *TGFB2*, and *TGFBR2* were all significantly upregulated in RNAMethyPro-identified high-risk tumors, we hypothesized that m^6^A regulators may functionally interact with EMT induced by activated TGFβ pathway in the stromal cells. This is in line with a recent study that showed TGFβ pathway as a major driver of m^6^A mRNA methylation.^41^

Although earlier studies have reported oncogenic and tumor-suppressive roles of different m^6^A regulators in various malignancies, no studies have yet been performed in CRC. Ours is the first comprehensive research interrogating association between m^6^A regulatory machinery and clinical outcomes in CRC. Clinically, in addition to demonstrating the robust prognostic value of RNAMethyPro, we also showed its association with anti-EGFR drug response in cell lines and metastatic CRC patients, though the statistical significance needs to be confirmed by further large-scale validations. In addition to facilitating selection of appropriate patients for anti-EGFR therapy, the ability to stratify cell lines for anti-EGFR response with allow us to test novel targets and drug combinations to sensitize the cell lines for anti-EGFR therapy and other novel treatments. Biologically, we found RNAMethyPro-stratified risk groups were significantly associated with MSI/MSS, CIMP status, *BRAF* mutations, and more importantly, CMSs of CRC. This is in line with previous biological findings, where FTO was shown to be associated with poor prognosis molecular subtypes of breast cancer and AML.

We would like to acknowledge that our findings are based on in silico analysis, which are critical for obtaining a global overview of the biological and clinical characteristics associated with m^6^A machinery, as well as in determining the specific functional modules dysregulated in high-risk patients. Further mechanistic and independent clinical validation studies are needed to validate the significance of RNAMethyPro as a robust prognostic and predictive signature in various human cancers.

In conclusion, we developed RNAMethyPro, a novel gene expression signature comprised of seven m^6^A regulators for prognosis in multiple cancers. Using comprehensive pan-cancer analysis, we identified activated EMT as a highly conserved biological process across multiple cancer types. Further investigation on CRC revealed the association of RNAMethyPro high-risk group with the mesenchymal subtype and poor anti-EGFR response. With future validation and in-depth mechanistic studies, RNAMethyPro may offer an important clinical decision-making tool in the future.

## Methods

### Development and validation of m^6^A prognostic classifiers

In order to develop m^6^A prognostic classifiers and evaluate the prognostic performance, we collected and analyzed a total of 9770 specimens, which comprised of 25 datasets for 13 different types of cancers (Table 1, Supplementary Material and Methods). For colorectal, gastric, breast, and ovarian cancers, we analyzed data from two independent patient cohorts for the internal and external validations. To make gene expression levels comparable, *z*-normalization was performed in each dataset. For each cancer type, a multivariate Cox regression model was trained on the corresponding training set, and the trained model was subsequently used to calculate risk scores for both the training and validation (if available) datasets. Patients were subsequently stratified into low-, intermediate-, and high-risk groups, using the 25th and 75th percentile risk scores derived from the training sets as the cutoff thresholds. To evaluate the prognostic performance, 5-year DFS was considered as an indicator for colorectal, gastric, and breast cancers, while OS was used for ovarian cancer due to limited clinical records and relatively short follow-up. For other cancers, the three risk groups were stratified using the same cutoff thresholds at 25th and 75th percentiles of risk scores, derived from the Cox regression model trained on the corresponding dataset. Only patients with valid survival information available were used in the analyses.

### Gene set enrichment analysis

Based on RNAMethyPro risk stratification, differentially expressed genes between low- and high-risk groups were identified based on TCGA datasets from 13 cancer types, using “LIMMA” R package. GSEA was performed using HTSanalyzeR^28^ with 5000 permutations for 50 hallmark gene sets (≥15 genes) obtained from MSigDB v6.1. To illustrate the association between these 50 hallmark gene sets, we constructed an enrichment map, where nodes encoded gene set size and edges encodes the strength of association quantified by Jaccard similarity coefficient (or Jaccard index). Node color represented conservation scores, defined by the frequency that a gene set is significantly enriched (*P* < 0.05) in the RNAMethyPro high-risk group in each of the cancer types studied.

### ESTIMATE analysis of stromal and immune content

In order to confirm the hypothesis that CRC patients in the RNAMethyPro high-risk group had higher stromal and immune content, gene expression profiles from TCGA-COADREAD cohort were used for calculating stromal and immune scores with ESTIMATE.^49^ The statistical significance of differences between the high- and intermediate-/low-risk groups were evaluated using Kruskal–Wallis tests.

### Network analysis

To identify functional modules dysregulated in the RNAMethyPro high-risk groups conserved across the ten cancer types (OV, HCC, LUSC, LUAD, HNSC, GC, ESCC, EAC, CRC, and BLCA), we employed BioNet, a model-based network approach previously published.^50^ Specifically, we aggregated *P* values derived from differential gene expression analysis using “LIMMA” R package between RNAMethyPro high- and low-risk groups in the ten cancer types by tenth order statistic. After successfully fitting the aggregated *P* values to a beta-uniform mixture model, signal-to-noise ratios were calculated to score gene products in the human interactome retrieved from BioGRID database (version 3.4.134), followed by identification of enriched subnetwork using “BioNet” R package^50^ (false discovery rate <1e−4). The obtained subnetwork of PPIs is visualized using “RedeR” R package.

### Statistical analysis

Statistical analyses were performed using R (version 3.4.3, www.r-project.org). Continuous variables were expressed as mean and standard error of the mean and were compared using Student’s *t* tests or Wilcoxon rank-sum tests. Categorical variables were compared using one-tailed Fisher’s exact tests or hypergeometric tests. Survival analyses were performed using the Kaplan–Meier method and compared with log-rank tests using “survival” package. Multivariate Cox regression models were trained using “coxph” function in “survival” package. HRs were calculated using function “hazard.ratio” in “survcomp” package. *P* < 0.05 was considered as significant for all tests.

### Reporting Summary

Further information on experimental design is available in the Nature Research Reporting Summary linked to this article.

## Supplementary information

Supplementary Material

Reporting Summary

## Data Availability

The authors declare that the data supporting our findings are all accessible from public repositories, and their accession codes can be found in Table 1.

## Supplementary information

**Supplementary information** accompanies the paper on the *npj Precision Oncology* website (10.1038/s41698-019-0085-2).
