# Supplementary material for: RNAMethyPro: a biologically conserved signature of N6-methyladenosine regulators for predicting survival at pan-cancer level
Source: NPJ Precis Oncol. 2019 May 1;3:13. doi: 10.1038/s41698-019-0085-2 (PMC6494854; doi:10.1038/s41698-019-0085-2)

**Supplemental material**

**RNAMethyPro: A biologically conserved signature of N6-methyladenosine regulators for predicting survival at pan-cancer level**

Raju Kandimalla^1*^, Feng Gao^2,4*^, Ying Li^2*^, Hao Huang^2^, Jia Ke^4^, Xin Deng^2^, Linjie Zhao^5^, Shengtao Zhou^5^, and Ajay Goel^1#^, Xin Wang^2,3,#^

**Affiliations:**

^1^ Center for Gastrointestinal Research; Center for Translational Genomics and Oncology, Baylor Scott & White Research Institute and Charles A Sammons Cancer Center, Baylor University Medical Center, Dallas, Texas, USA

^2^ Department of Biomedical Sciences, City University of Hong Kong, Hong Kong, China

^3^ Shenzhen Research Institute, City University of Hong Kong, China

^4^ The Sixth Affiliated Hospital of Sun Yat-Sen University, Guangzhou, China

^5^ Department of Obstetrics and Gynecology, Key Laboratory of Birth Defects and Related Diseases of Women and Children of MOE and State Key Laboratory of Biotherapy, West China Second University Hospital, Sichuan University and Collaborative Innovation Center, Chengdu, China

**^*^ These authors contributed equally to this work:** Raju Kandimalla, Feng Gao, Ying Li

**^#^Correspondence:** xin.wang@cityu.edu.hk (X.W.); Ajay.Goel@BSWHealth.org (A.G.)

## Material and Methods

#### Public datasets

In this study, we analyzed a total of 9,770 specimens, which comprised of 25 datasets for 13 different types of cancers (**Table 1**). TCGA datasets, level-3 gene expression profiles for tumor specimens, were downloaded from Firehose Broad GDAC portal (<http://gdac.broadinstitute.org/>, accessed on Jun 1, 2017) for colorectal adenocarcinomas (TCGA-COADREAD[^1^](https://paperpile.com/c/1Ge8Ih/oBVOP), *n* = 626), stomach adenocarcinomas (TCGA-STAD[^2^](https://paperpile.com/c/1Ge8Ih/csz9b), *n* = 415), pancreatic ductal adenocarcinomas (TCGA-PAAD[^3^](https://paperpile.com/c/1Ge8Ih/CBq57), *n* = 179), liver hepatocellular carcinomas (TCGA-LIHC[^4^](https://paperpile.com/c/1Ge8Ih/0uCBn), *n* = 373), ovarian serous cystadenocarcinomas (TCGA-OV[^5^](https://paperpile.com/c/1Ge8Ih/9ONRs), *n* = 514), lung adenocarcinomas (TCGA-LUAD[^6^](https://paperpile.com/c/1Ge8Ih/4IMDY), *n* = 517), lung squamous cell carcinomas (TCGA-LUSC[^7^](https://paperpile.com/c/1Ge8Ih/z242z), *n* = 501), esophageal carcinomas (TCGA-ESCA[^8^](https://paperpile.com/c/1Ge8Ih/St9fU), *n* = 165, including esophageal squamous cell carcinoma and esophageal adenocarcinoma), head and neck squamous cell carcinoma (TCGA-HNSC[^9^](https://paperpile.com/c/1Ge8Ih/TjqUj), *n* = 522), bladder urothelial carcinoma (TCGA-BLCA[^10^](https://paperpile.com/c/1Ge8Ih/6mhqz), *n* = 408) and breast cancer (TCGA-BRCA[^11^](https://paperpile.com/c/1Ge8Ih/Kn4me), *n* = 1100) respectively. For all the datasets other than the ovarian cohort, RSEM scaled estimates of gene expression levels were first converted to transcripts per million (TPM) by multiplication with 1 million, followed by log2-transformation. The TCGA-OV dataset consisted of processed gene expression profiles based on Affymetrix HG133A microarrays. For TCGA-PAAD dataset, we kept 76 specimens of high purity for further analysis. For TCGA-BRCA dataset, we analyzed 517 specimens with complete clinical information.

From Gene Expression Omnibus (GEO) database, we also obtained CIT (GSE39582[^12^](https://paperpile.com/c/1Ge8Ih/lvMO6), *n* = 566), Khambata-Ford (GSE5851[^13^](https://paperpile.com/c/1Ge8Ih/LrBpJ), *n* = 80), Jorissen (GSE14333[^14^](https://paperpile.com/c/1Ge8Ih/nvlwI), *n* = 290), Smith (GSE17536[^15^](https://paperpile.com/c/1Ge8Ih/JYHPO), *n* = 177), Birnbaum (GSE26906[^16^](https://paperpile.com/c/1Ge8Ih/fSGx1), *n* = 86), AMC-AJCCII-90 (GSE33113[^17^](https://paperpile.com/c/1Ge8Ih/8FdoJ), *n* = 90), Laibe (GSE37892[^18^](https://paperpile.com/c/1Ge8Ih/UQj3A), *n* = 130), Kirzin (GSE39084[^19^](https://paperpile.com/c/1Ge8Ih/yRmNG), *n* = 68), Medico (GSE59857[^20^](https://paperpile.com/c/1Ge8Ih/OpVnU), n = 155, of which 151 with cetuximab response) datasets for colorectal cancer, ACRG-GC dataset (GSE62254[^21^](https://paperpile.com/c/1Ge8Ih/iNzZc), *n* = 300) for gastric cancer, as well as MAYO-OV dataset (GSE53963[^22^](https://paperpile.com/c/1Ge8Ih/ewi1n), *n* = 174) for ovarian cancer in their processed form. GSE39582, GSE14333, GSE17536, GSE26906, GSE33113, GSE37892, GSE39084, GSE5851, GSE62254 were all based on Affymetrix Human Genome U133 Plus 2.0 Arrays, and the probe set IDs were converted to official gene symbols according to the annotation ‘GPL570’ in GEO. GSE59857 dataset was measured on Illumina HumanHT-12 V4.0 expression beadchip platform and annotated by ‘GPL10558’ in GEO. All datasets from GEO database were downloaded directly in their processed form. For 7 GEO datasets used as the CRC meta-validation set, we further removed the non-biological batch effects using ‘combat’ function in R ‘sva’ package.

Additionally, METABRIC[^23^](https://paperpile.com/c/1Ge8Ih/wEUqk) gene expression discovery and validation datasets were obtained for breast cancer analysis. For acute myeloid leukemia (AML), RNA-seq data was downloaded from TARGET[^24^](https://paperpile.com/c/1Ge8Ih/zpFl3) database, and was first converted from FPKM to TPM followed by log2-transformation. CRC cell line datasets were obtained from Cancer Cell Line Encyclopedia (CCLE)[^25^](https://paperpile.com/c/1Ge8Ih/wfAI). More specifically, RNA-seq gene expression data (CCLE_RNAseq_081117.rpkm.gct) was downloaded from *http://www.broadinstitute.org/ccle*, and was first converted from RPKM to TPM followed by log2-transformation.

For genes with multiple probe sets, we kept the ones with the largest median absolute deviations (MADs). Only patients with complete survival information were used for survival analyses. In the TCGA-OV dataset, two genes (*METTL14* and *ALKBH5*) were missing, and therefore, we marked corresponding values as ‘*NA*’. For all colorectal cancer datasets, CMS (consensus molecular subtype) labels were calculated using ‘CMSclassifier’ package (https://github.com/Sage-Bionetworks/CMSclassifier).

## References

1. [Cancer Genome Atlas Network. Comprehensive molecular characterization of human colon and rectal cancer. *Nature* **487**, 330–337 (2012).](http://paperpile.com/b/1Ge8Ih/oBVOP)

2. [Cancer Genome Atlas Research Network. Comprehensive molecular characterization of gastric adenocarcinoma. *Nature* **513**, 202–209 (2014).](http://paperpile.com/b/1Ge8Ih/csz9b)

3. [Cancer Genome Atlas Research Network. Electronic address: andrew_aguirre@dfci.harvard.edu & Cancer Genome Atlas Research Network. Integrated Genomic Characterization of Pancreatic Ductal Adenocarcinoma. *Cancer Cell* **32**, 185–203.e13 (2017).](http://paperpile.com/b/1Ge8Ih/CBq57)

4. [Cancer Genome Atlas Research Network. Electronic address: wheeler@bcm.edu & Cancer Genome Atlas Research Network. Comprehensive and Integrative Genomic Characterization of Hepatocellular Carcinoma. *Cell* **169**, 1327–1341.e23 (2017).](http://paperpile.com/b/1Ge8Ih/0uCBn)

5. [Cancer Genome Atlas Research Network. Integrated genomic analyses of ovarian carcinoma. *Nature* **474**, 609–615 (2011).](http://paperpile.com/b/1Ge8Ih/9ONRs)

6. [Cancer Genome Atlas Research Network. Comprehensive molecular profiling of lung adenocarcinoma. *Nature* **511**, 543–550 (2014).](http://paperpile.com/b/1Ge8Ih/4IMDY)

7. [Cancer Genome Atlas Research Network. Comprehensive genomic characterization of squamous cell lung cancers. *Nature* **489**, 519–525 (2012).](http://paperpile.com/b/1Ge8Ih/z242z)

8. [Cancer Genome Atlas Research Network *et al.* Integrated genomic characterization of oesophageal carcinoma. *Nature* **541**, 169–175 (2017).](http://paperpile.com/b/1Ge8Ih/St9fU)

9. [Cancer Genome Atlas Network. Comprehensive genomic characterization of head and neck squamous cell carcinomas. *Nature* **517**, 576–582 (2015).](http://paperpile.com/b/1Ge8Ih/TjqUj)

10. [Cancer Genome Atlas Research Network. Comprehensive molecular characterization of urothelial bladder carcinoma. *Nature* **507**, 315–322 (2014).](http://paperpile.com/b/1Ge8Ih/6mhqz)

11. [Ciriello, G. *et al.* Comprehensive Molecular Portraits of Invasive Lobular Breast Cancer. *Cell* **163**, 506–519 (2015).](http://paperpile.com/b/1Ge8Ih/Kn4me)

12. [Marisa, L. *et al.* Gene expression classification of colon cancer into molecular subtypes: characterization, validation, and prognostic value. *PLoS Med.* **10**, e1001453 (2013).](http://paperpile.com/b/1Ge8Ih/lvMO6)

13. [Khambata-Ford, S. *et al.* Expression of epiregulin and amphiregulin and K-ras mutation status predict disease control in metastatic colorectal cancer patients treated with cetuximab. *J. Clin. Oncol.* **25**, 3230–3237 (2007).](http://paperpile.com/b/1Ge8Ih/LrBpJ)

14. [Jorissen, R. N. *et al.* Metastasis-Associated Gene Expression Changes Predict Poor Outcomes in Patients with Dukes Stage B and C Colorectal Cancer. *Clin. Cancer Res.* **15**, 7642–7651 (2009).](http://paperpile.com/b/1Ge8Ih/nvlwI)

15. [Smith, J. J. *et al.* Experimentally derived metastasis gene expression profile predicts recurrence and death in patients with colon cancer. *Gastroenterology* **138**, 958–968 (2010).](http://paperpile.com/b/1Ge8Ih/JYHPO)

16. [Birnbaum, D. J. *et al.* Expression Profiles in Stage II Colon Cancer According to APC Gene Status. *Transl. Oncol.* **5**, 72–76 (2012).](http://paperpile.com/b/1Ge8Ih/fSGx1)

17. [de Sousa E Melo, F. *et al.* Methylation of Cancer-Stem-Cell-Associated Wnt Target Genes Predicts Poor Prognosis in Colorectal Cancer Patients. *Cell Stem Cell* **9**, 476–485 (2011).](http://paperpile.com/b/1Ge8Ih/8FdoJ)

18. [Laibe, S. *et al.* A seven-gene signature aggregates a subgroup of stage II colon cancers with stage III. *OMICS* **16**, 560–565 (2012).](http://paperpile.com/b/1Ge8Ih/UQj3A)

19. [Kirzin, S. *et al.* Sporadic early-onset colorectal cancer is a specific sub-type of cancer: a morphological, molecular and genetics study. *PLoS One* **9**, e103159 (2014).](http://paperpile.com/b/1Ge8Ih/yRmNG)

20. [Medico, E. *et al.* The molecular landscape of colorectal cancer cell lines unveils clinically actionable kinase targets. *Nat. Commun.* **6**, 7002 (2015).](http://paperpile.com/b/1Ge8Ih/OpVnU)

21. [Cristescu, R. *et al.* Molecular analysis of gastric cancer identifies subtypes associated with distinct clinical outcomes. *Nat. Med.* **21**, 449–456 (2015).](http://paperpile.com/b/1Ge8Ih/iNzZc)

22. [Konecny, G. E. *et al.* Prognostic and therapeutic relevance of molecular subtypes in high-grade serous ovarian cancer. *J. Natl. Cancer Inst.* **106**, (2014).](http://paperpile.com/b/1Ge8Ih/ewi1n)

23. [Curtis, C. *et al.* The genomic and transcriptomic architecture of 2,000 breast tumours reveals novel subgroups. *Nature* **486**, 346–352 (2012).](http://paperpile.com/b/1Ge8Ih/wEUqk)

24. [Farrar, J. E. *et al.* Genomic Profiling of Pediatric Acute Myeloid Leukemia Reveals a Changing Mutational Landscape from Disease Diagnosis to Relapse. *Cancer Res.* **76**, 2197–2205 (2016).](http://paperpile.com/b/1Ge8Ih/zpFl3)

25. [Barretina, J. *et al.* The Cancer Cell Line Encyclopedia enables predictive modelling of anticancer drug sensitivity. *Nature* **483**, 603–607 (2012).](http://paperpile.com/b/1Ge8Ih/wfAI)

## Supplementary Figures and Tables

**Figure S1** Internal validation of the prognostic value of RNAMethyPro in the other 9 cancer types. Kaplan-Meier graphs stratified for RNAMethyPro risk groups for (**a**) Pancreatic adenocarcinoma (TCGA-PAAD cohort, *n* = 149); (**b**) Hepatocellular carcinoma (TCGA-LIHC cohort, *n* = 287); (**c**) TCGA Lung adenocarcinoma (TCGA-LUAD cohort, *n* = 249); (**d**) Bladder urothelial carcinoma (TCGA-BLCA cohort, *n* = 275); (**e**) Head and Neck squamous cell carcinoma (TCGA-HNSC cohort, *n* = 261); (**f**) Acute myeloid leukemia (TARGET-AML cohort, *n* = 284); (**g**) Lung squamous cell carcinoma (TCGA-LUSC cohort, *n* = 154); (**h**) Esophageal adenocarcinoma (TCGA-ESCA (EAC) cohort, *n* = 32); (**i**) Esophageal squamous cell carcinoma (ECGA-ESCA (ESCC) cohort, *n* = 70). Only patients with available survival information were included in the analyses.

**Figure S2** GSEA plots for EMT in 10 cancer types.

**Figure S3** GSEA plots for characteristic gene signatures and pathways associated with the mesenchymal CMS4 subtype of CRC (TCGA-COADREAD cohort, *n* = 626).

**Figure S4** Bar plots compare normalized expression levels of EMT signature genes in both (a) cell lines and (c) CRC tissue samples between different risk groups predicted using RNAMethyPro. Statistical significance of differential expression between RNAMethyPro high- and low-risk groups was estimated by one-tailed Student’s t-tests. (n=16 for low-risk group, n=33 for intermediate-risk group, n=9 for high-risk group in cell lines. n=445 for low-risk group, n=563 for intermediate-risk group, n=398 for high-risk group in CRC tissue samples. Error bar: standard error of the mean). Gene set enrichment analyses illustrate significant upregulation of EMT program in (b) cell lines and (d) CRC tissue samples classified to the high-risk groups (*P* < 0.001).

**Figure S5** A coexpression network of functional associations between m^6^A regulator genes (red nodes), hub genes in the conserved subnetwork (green nodes) and EMT signature genes (blue nodes). Edge widths are proportionate to Pearson correlation coefficients between gene expression levels of gene pairs that are significantly correlated (*P* < 0.05) in the CRC META-validation cohort.

**Table S1** Pearson correlation coefficients and associated p-values quantifying the strength and statistical significance of coexpression between the seven m^6^A regulator genes, EMT signature genes (*ZEB1*, *SMAD2*, *SMAD3*, *TGFB2*, *TGFBR2*) and the four hub genes (*XPO1*, *NTRK1*, *ELAVL1* and *APP*)

**Table S2** Univariate and multivariate analysis of RNAMethyPro risk score, and available molecular and clinical factors in the Khambata-Ford cohort

**Table S1**

| **Gene 1** | **Gene 2** | **Pearson Correlation Coefficient** | **P-value of correlation** |
| --- | --- | --- | --- |
| YTHDF2 | YTHDF1 | -0.053 | 0.211 |
| ALKBH5 | YTHDF1 | -0.296 | <0.001 |
| ALKBH5 | YTHDF2 | 0.278 | <0.001 |
| FTO | YTHDF1 | 0.198 | <0.001 |
| FTO | YTHDF2 | 0.027 | 0.518 |
| FTO | ALKBH5 | 0.116 | 0.006 |
| METTL3 | YTHDF1 | -0.044 | 0.301 |
| METTL3 | YTHDF2 | 0.228 | <0.001 |
| METTL3 | ALKBH5 | 0.139 | 0.001 |
| METTL3 | FTO | -0.105 | 0.012 |
| METTL14 | YTHDF1 | -0.079 | 0.061 |
| METTL14 | YTHDF2 | 0.177 | <0.001 |
| METTL14 | ALKBH5 | -0.024 | 0.567 |
| METTL14 | FTO | -0.178 | <0.001 |
| METTL14 | METTL3 | 0.091 | 0.031 |
| WTAP | YTHDF1 | 0.013 | 0.766 |
| WTAP | YTHDF2 | 0.135 | 0.001 |
| WTAP | ALKBH5 | 0.013 | 0.752 |
| WTAP | FTO | 0.05 | 0.237 |
| WTAP | METTL3 | -0.032 | 0.445 |
| WTAP | METTL14 | 0.111 | 0.008 |
| ZEB1 | YTHDF1 | -0.125 | 0.003 |
| ZEB1 | YTHDF2 | -0.133 | 0.002 |
| ZEB1 | ALKBH5 | 0.003 | 0.941 |
| ZEB1 | FTO | 0.323 | <0.001 |
| ZEB1 | METTL3 | -0.085 | 0.044 |
| ZEB1 | METTL14 | 0.183 | <0.001 |
| ZEB1 | WTAP | 0.174 | <0.001 |
| SMAD2 | YTHDF1 | -0.306 | <0.001 |
| SMAD2 | YTHDF2 | 0.272 | <0.001 |
| SMAD2 | ALKBH5 | 0.367 | <0.001 |
| SMAD2 | FTO | 0.035 | 0.406 |
| SMAD2 | METTL3 | 0.118 | 0.005 |
| SMAD2 | METTL14 | 0.246 | <0.001 |
| SMAD2 | WTAP | 0.373 | <0.001 |
| SMAD2 | ZEB1 | 0.208 | <0.001 |
| SMAD3 | YTHDF1 | 0.119 | 0.005 |
| SMAD3 | YTHDF2 | 0.058 | 0.167 |
| SMAD3 | ALKBH5 | 0.091 | 0.031 |
| SMAD3 | FTO | 0.118 | 0.005 |
| SMAD3 | METTL3 | -0.082 | 0.051 |
| SMAD3 | METTL14 | 0.054 | 0.204 |
| SMAD3 | WTAP | 0.029 | 0.489 |
| SMAD3 | ZEB1 | 0.283 | <0.001 |
| SMAD3 | SMAD2 | 0.086 | 0.041 |
| TGFB2 | YTHDF1 | -0.059 | 0.161 |
| TGFB2 | YTHDF2 | -0.03 | 0.477 |
| TGFB2 | ALKBH5 | 0.044 | 0.294 |
| TGFB2 | FTO | 0.23 | <0.001 |
| TGFB2 | METTL3 | -0.014 | 0.736 |
| TGFB2 | METTL14 | 0.177 | <0.001 |
| TGFB2 | WTAP | 0.134 | 0.001 |
| TGFB2 | ZEB1 | 0.546 | <0.001 |
| TGFB2 | SMAD2 | 0.185 | <0.001 |
| TGFB2 | SMAD3 | 0.221 | <0.001 |
| TGFBR2 | YTHDF1 | 0.028 | 0.511 |
| TGFBR2 | YTHDF2 | -0.12 | 0.004 |
| TGFBR2 | ALKBH5 | -0.194 | <0.001 |
| TGFBR2 | FTO | 0.097 | 0.021 |
| TGFBR2 | METTL3 | 0.025 | 0.555 |
| TGFBR2 | METTL14 | 0.212 | <0.001 |
| TGFBR2 | WTAP | 0.108 | 0.01 |
| TGFBR2 | ZEB1 | 0.342 | <0.001 |
| TGFBR2 | SMAD2 | 0.044 | 0.293 |
| TGFBR2 | SMAD3 | 0.036 | 0.39 |
| TGFBR2 | TGFB2 | 0.271 | <0.001 |
| XPO1 | YTHDF1 | 0.304 | <0.001 |
| XPO1 | YTHDF2 | 0.131 | 0.002 |
| XPO1 | ALKBH5 | -0.17 | <0.001 |
| XPO1 | FTO | -0.028 | 0.511 |
| XPO1 | METTL3 | 0.212 | <0.001 |
| XPO1 | METTL14 | 0.314 | <0.001 |
| XPO1 | WTAP | 0.385 | <0.001 |
| XPO1 | ZEB1 | 0.066 | 0.12 |
| XPO1 | SMAD2 | 0.183 | <0.001 |
| XPO1 | SMAD3 | 0.071 | 0.091 |
| XPO1 | TGFB2 | 0.072 | 0.086 |
| XPO1 | TGFBR2 | 0.09 | 0.032 |
| NTRK1 | YTHDF1 | -0.007 | 0.874 |
| NTRK1 | YTHDF2 | -0.146 | <0.001 |
| NTRK1 | ALKBH5 | -0.009 | 0.84 |
| NTRK1 | FTO | -0.002 | 0.971 |
| NTRK1 | METTL3 | -0.035 | 0.412 |
| NTRK1 | METTL14 | -0.312 | <0.001 |
| NTRK1 | WTAP | -0.274 | <0.001 |
| NTRK1 | ZEB1 | -0.12 | 0.004 |
| NTRK1 | SMAD2 | -0.299 | <0.001 |
| NTRK1 | SMAD3 | -0.112 | 0.008 |
| NTRK1 | TGFB2 | -0.164 | <0.001 |
| NTRK1 | TGFBR2 | -0.061 | 0.147 |
| NTRK1 | XPO1 | -0.376 | <0.001 |
| ELAVL1 | YTHDF1 | 0.278 | <0.001 |
| ELAVL1 | YTHDF2 | 0.24 | <0.001 |
| ELAVL1 | ALKBH5 | 0.102 | 0.015 |
| ELAVL1 | FTO | 0.325 | <0.001 |
| ELAVL1 | METTL3 | 0.039 | 0.358 |
| ELAVL1 | METTL14 | -0.185 | <0.001 |
| ELAVL1 | WTAP | 0.144 | 0.001 |
| ELAVL1 | ZEB1 | -0.25 | <0.001 |
| ELAVL1 | SMAD2 | 0.044 | 0.296 |
| ELAVL1 | SMAD3 | -0.058 | 0.165 |
| ELAVL1 | TGFB2 | -0.092 | 0.028 |
| ELAVL1 | TGFBR2 | -0.156 | <0.001 |
| ELAVL1 | XPO1 | 0.294 | <0.001 |
| ELAVL1 | NTRK1 | -0.16 | <0.001 |
| APP | YTHDF1 | -0.084 | 0.047 |
| APP | YTHDF2 | 0.03 | 0.471 |
| APP | ALKBH5 | -0.1 | 0.017 |
| APP | FTO | 0.088 | 0.037 |
| APP | METTL3 | -0.112 | 0.008 |
| APP | METTL14 | 0.104 | 0.013 |
| APP | WTAP | 0.026 | 0.544 |
| APP | ZEB1 | 0.068 | 0.108 |
| APP | SMAD2 | 0.163 | <0.001 |
| APP | SMAD3 | -0.051 | 0.231 |
| APP | TGFB2 | 0.133 | 0.002 |
| APP | TGFBR2 | 0.123 | 0.003 |
| APP | XPO1 | 0.035 | 0.403 |
| APP | NTRK1 | -0.175 | <0.001 |
| APP | ELAVL1 | 0.055 | 0.189 |

**Table S2**

|  | **Univariate analysis** | | | |
| --- | --- | --- | --- | --- |
|  | **HR** | **95% CI low** | **95% CI high** | ***P*** |
| Age | 1.00 | 0.99 | 1.01 | 0.93 |
| KRAS | 1.34 | 1.10 | 1.57 | 0.21 |
| RNAMethyPro risk score | 1.82 | 1.53 | 2.11 | 0.04 |
|  | **Multivariate analysis** | | | |
|  | **HR** | **95% CI low** | **95% CI high** | ***P*** |
| Age | 1.00 | 0.99 | 1.01 | 0.80 |
| KRAS | 1.38 | 1.14 | 1.62 | 0.18 |
| RNAMethyPro risk score | 1.86 | 1.57 | 2.14 | 0.03 |

**Figure S1:**


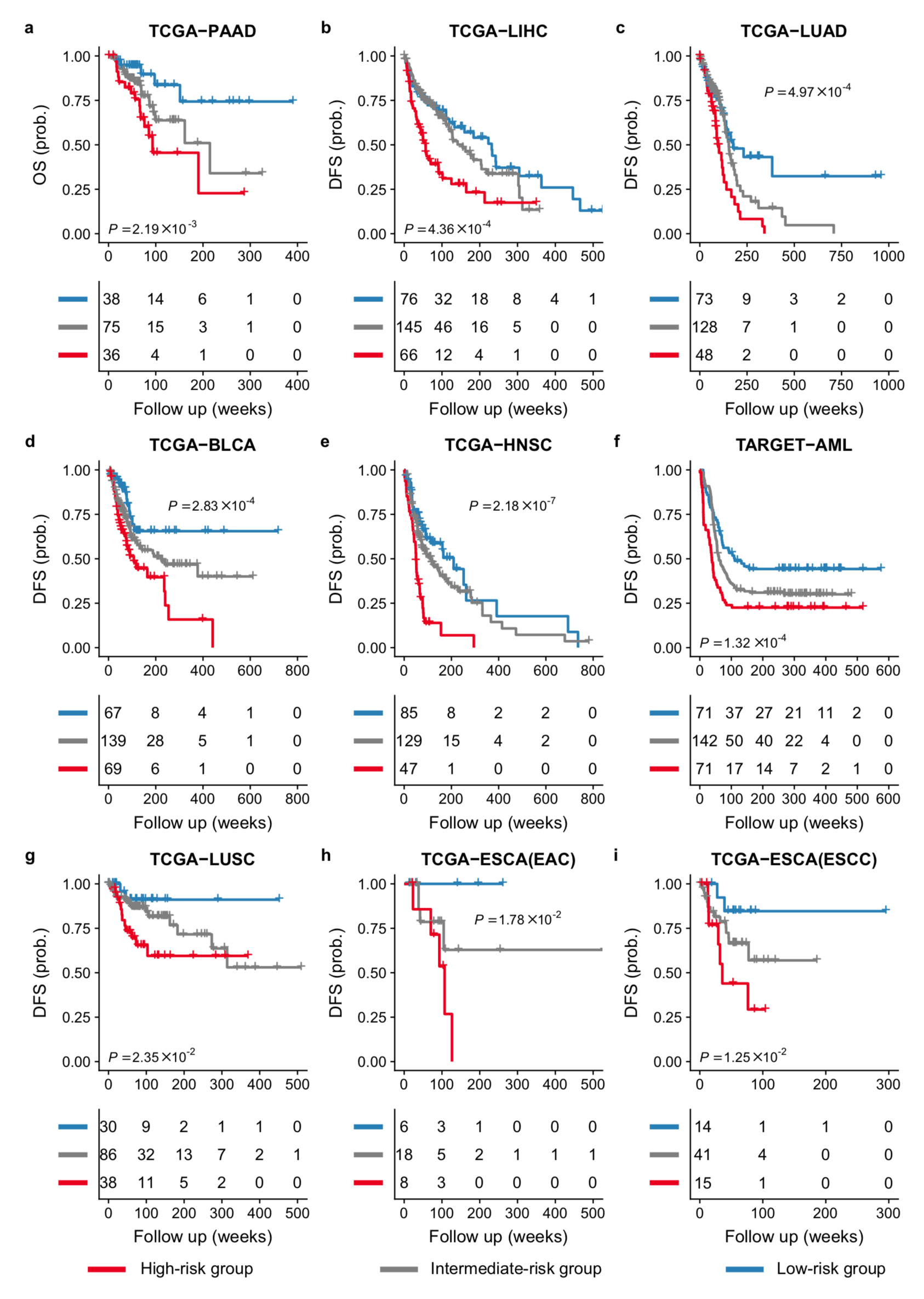


**Figure S2:**


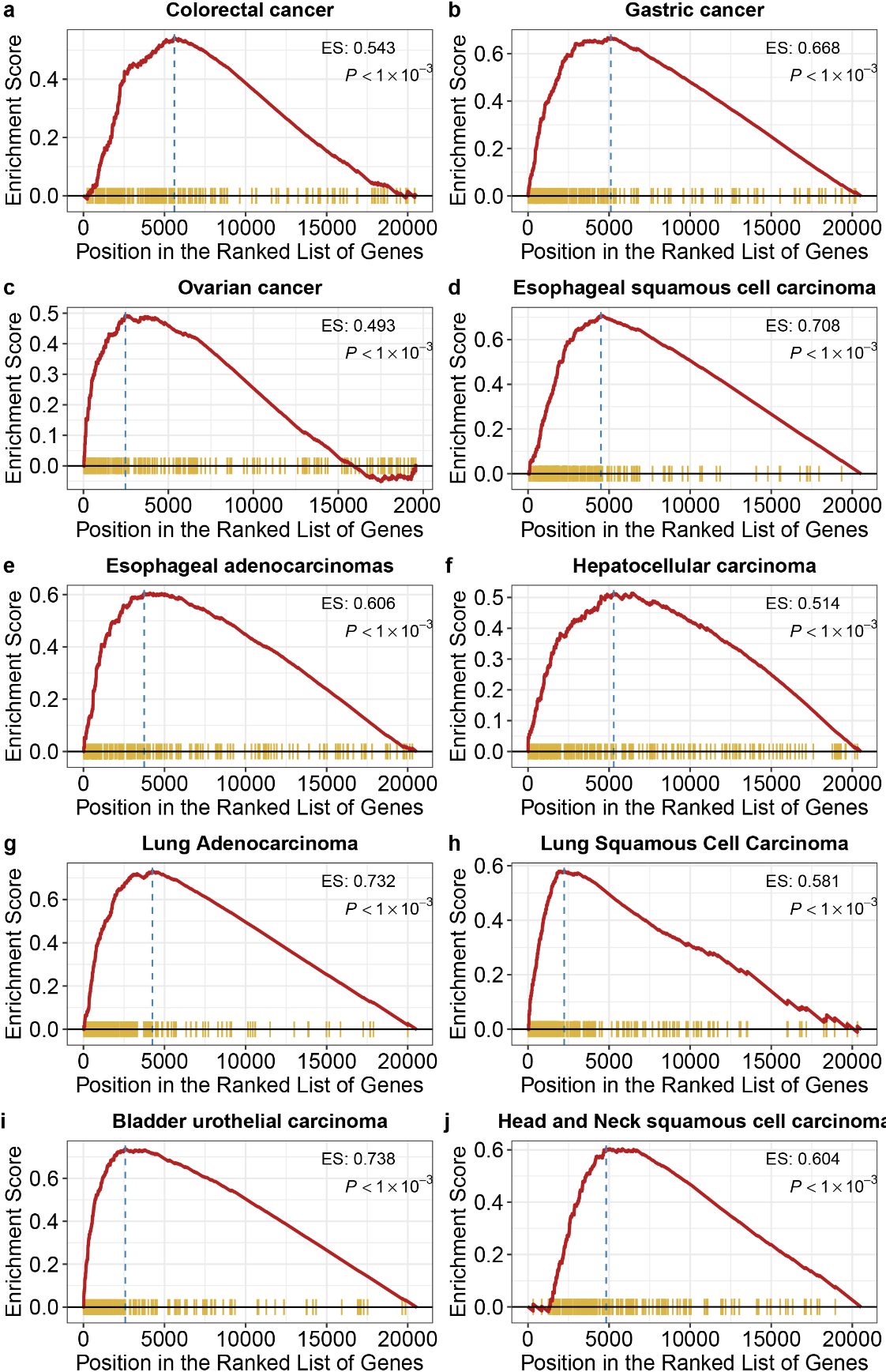


**Figure S3:**


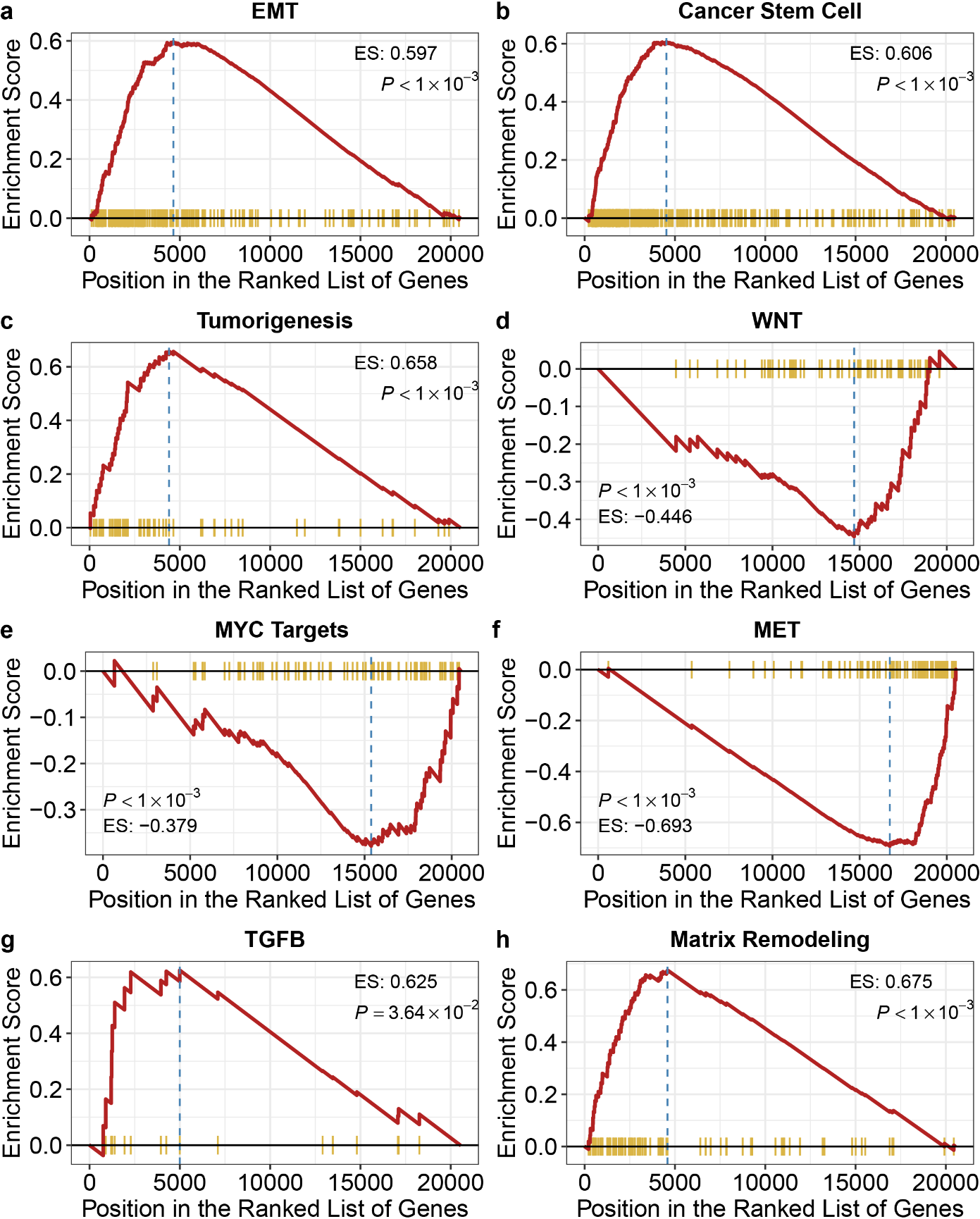


**Figure S4:**


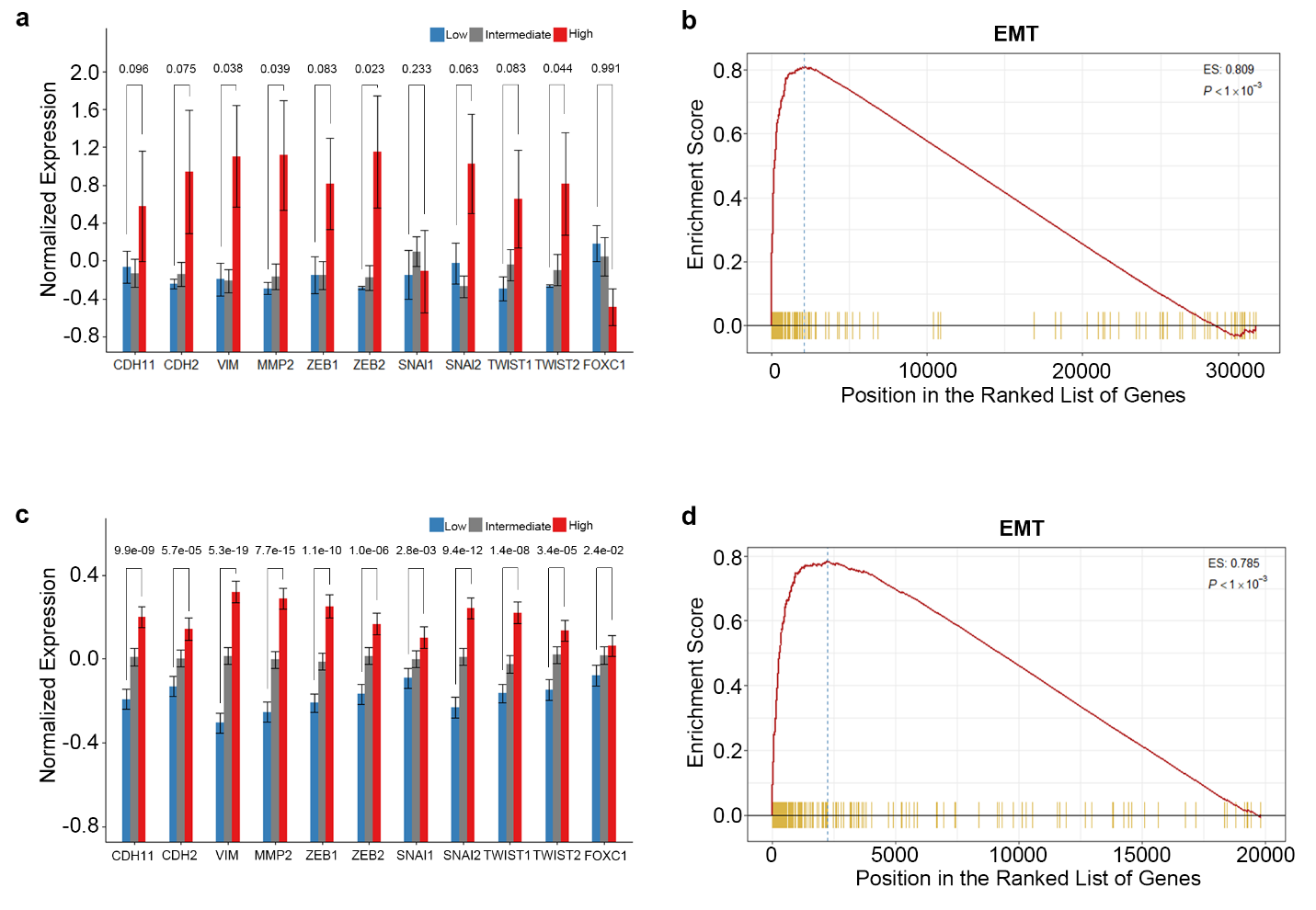


**Figure S5:**


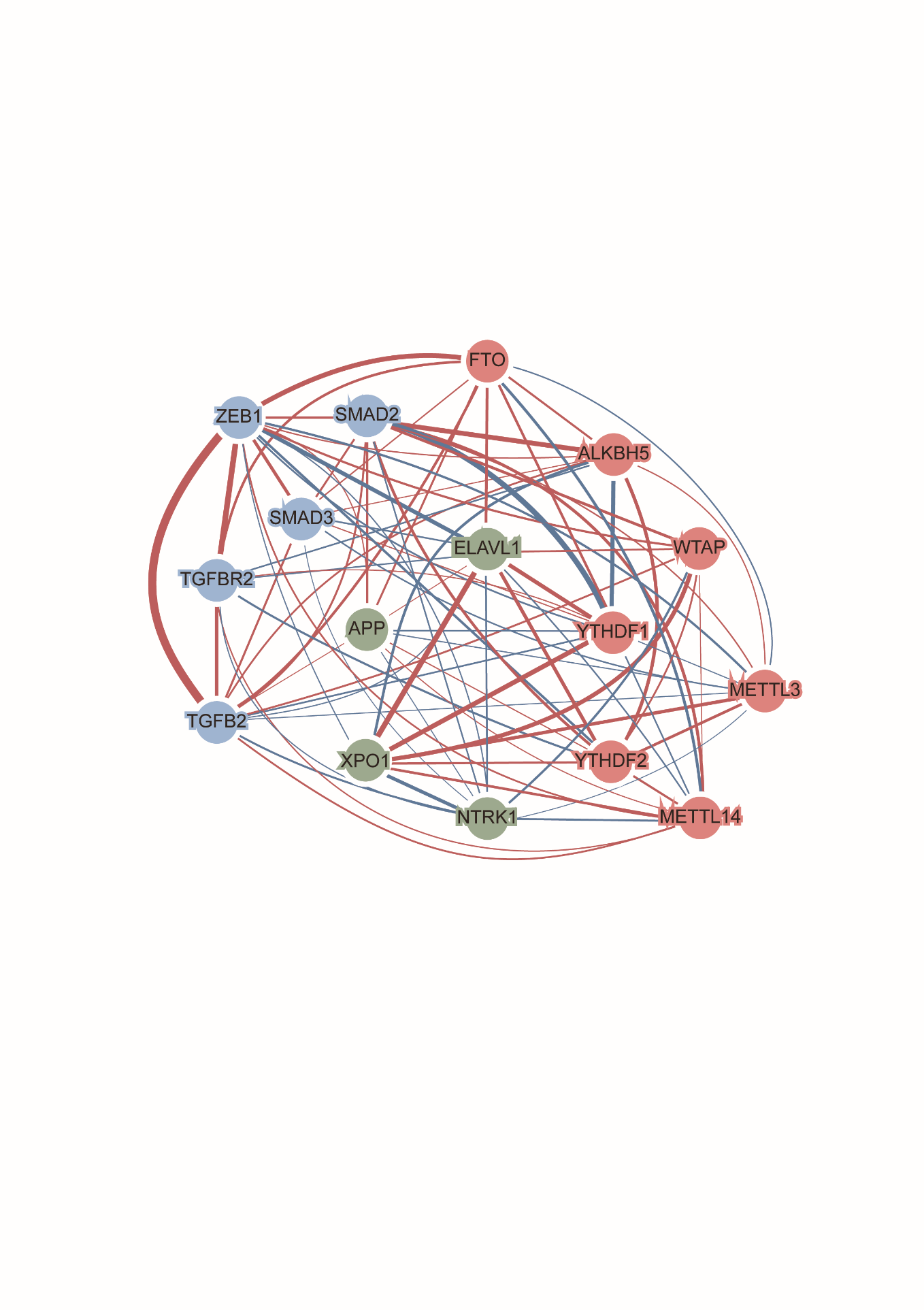

Supplement: Supplementary file 1 — Supplementary Material [file 41698_2019_85_MOESM1_ESM.docx]
